# Separation of hydrocarbons from activated carbon as a porous substance in a glycol regeneration process using supercritical carbon dioxide

**DOI:** 10.1038/s41598-022-23722-8

**Published:** 2022-11-19

**Authors:** Mohammad Najafi, Zahra Arab Abousadi, Bizhan Honarvar, Seyed Ali Sajadian

**Affiliations:** 1 Department of Chemical Engineering, Islamic Azad University of Marvdasht, Marvdasht, Iran; 2 South Zagros Oil and Gas Production, National Iranian Oil Company, Shiraz, 7135717991 Iran; 3 Department of Chemical Engineering, Faculty of Engineering, University of Kashan, Kashan, 87317-53153 Iran

**Keywords:** Chemical engineering, Chemistry, Engineering

## Abstract

Activated carbons are used in industrial applications; their cost is a major barrier to their more widespread application. Regeneration of activated carbons is indispensable to minimize operational costs and product wastage. Supercritical carbon dioxide (SC-CO_2_) as green technology was used to regenerate activated carbons. In this work, response surface methodology was employed to optimize the supercritical regeneration process and to evaluate the effect of the operational parameters including pressure (100–300 bar), temperature (313–333 K), flow rate (2–6 g/min), and dynamic time (30–150 min) on the regeneration yield. The maximum regeneration yield (93.71%) was achieved at 285 bar, 333 K, 4 g/min, and 147 min. Mathematical modeling was done using two one-parameter kinetics models, which agree well with the experimental data. The fitting parameter of the model was obtained by using a differential evolution algorithm. The chemical composition of the substances extracted from the activated carbon was identified by gas chromatography. The results showed that the regeneration of activated carbon by SC-CO_2_ can be an alternative method to conventional methods.

## Introduction

Activated carbon has been recognized as one of the widely applied adsorbents for spray coating^1^, food processing^2^, biomass^3^, pharmaceuticals^4^, chemicals^5^, waste water treatment^6^ petroleum^7^, and nuclear industries^8^, as well as the glycol treatment for removing organic and volatile organic compounds (VOCs) pollutants in natural gas industries^9^. Activated carbon is used in natural gas sweetening and dehydration systems with fluids such as amines and glycols^10,11^. The conventional techniques for the regeneration of activated carbon include thermal volatilization^12^, chemical extraction^13^, ultrasound^14^, microwave^15^, electrochemical^16^, and bio-regeneration. These methods have several disadvantages such as loss of carbon, damage of its porous structure , treatment of exhaust gases, chemical regenerations using solvents are not necessarily acceptable because additional separation and environmental problems and bio-regeneration requires long reaction time for regeneration. Recently, supercritical fluids (SCFs) has attracted widespread attention in many fields and regeneration of activated carbon as one of the applications of this technology has been studied^17^. The unique characteristics of SCFs have made these solvents attractive. Particularly, solvent density, and hence its dissolvent properties, can be controlled modifying pressure and temperature. Besides, liquid-like density and gas-like viscosity, coupled with diffusion coefficients that are at least an order of magnitude higher than those of liquids, contribute to the enhancement of the mass transfer processes^18–23^. Among the different substances, carbon dioxide is the best choice as it is an environment-friendly solvent providing such advantages as nontoxicity and high chemical stability.

Regeneration of activated carbon using SCFs has been studied by several researchers. DeFilippi et al. observed that the supercritical regeneration was economical even though the operating temperature and pressure were above 387 K and 150 atm, respectively. They proposed a local equilibrium model (Freundlich isotherm) fitting well with the experimental data^24^. Regeneration of activated carbon loaded with phenol using supercritical carbon dioxide was studied by Kander and Paulaitis^25^. They found that supercritical carbon dioxide offered no significant advantages for the regeneration of carbon loaded with phenol. However, they suggested that for organic compounds which are not adsorbed strongly onto activated carbon, supercritical carbon dioxide would be a powerful adsorbent. Tan and Liou investigated desorption by supercritical carbon dioxide of activated carbon loaded with either ethyl acetate or toluene. They presented that this regeneration method would give better results than the steam regeneration method and accordingly, presented a linear desorption kinetics model that was found to fit experimental data quite well^17,26^.

Madras et al.^27^ studied the desorption of several nonvolatile solids such as hexachlorobenzene and pentachlorophenol via break through experiments at a fixed carbon dioxide density. Macnaughton and Foster^28^ measured the adsorption equilibrium of 1,1,1-trichloro-2,2-bis (p-chlorophenyl) ethane (DDT) on an activated carbon and illustrated that one limiting factor on supercritical desorption process is adsorption equilibrium at a low CO_2_ flow rate. Using a three-parameter mode, the adsorption breakthrough and equilibrium of ethylbenzene on an activated carbon from supercritical carbon dioxide was investigated by Harikrishnan et al.^29^. Tan and Liou^26^ studied desorption behavior of ethyl acetate from activated carbon in CO_2_ at temperatures from 300 to 338 K and pressures from 8.7 to 12.9 MPa. They observed that increasing pressure and decreasing temperature increased the desorption rate. Benkhedda et al.^30^ also presented similar temperature and pressure dependence for m-xylene desorption behavior using SC-CO_2_ at temperatures from 313 to 333 K and pressures from 10.0 to 15.0 MPa. They suggested that desorption behavior of VOCs from activated carbons using SC-CO_2_ largely depends on temperature and pressure conditions. Salvador et al.^31^ studied the regeneration of activated carbons contaminated with phenol using supercritical water at 260 bar and temperature ranging between 400 and 500 °C. Regeneration temperature and time were the variables that most affected the process. Ikuo Ushiki et al.^9^ investigated the desorption behavior of various VOCs (toluene, acetone, n-hexane, n-octane, methanol, ethanol, 2-propanol, and propylene glycol monomethyl ether) from activated carbon using a fixed-bed method employing SC-CO_2_ at temperature values ranging between 313 and 353 K and pressure values ranging between 10.0 and 15.0 MPa.

Several factors such as pressure, temperature, CO_2_ flow rate, time, particle size, among others, simultaneous influenced the supercritical regeneration of activated carbons. In most of the previously published studies, the process conditions have just been optimized by conducting one-factor- at-a-time experiments. The results of one-factor-at-a-time experiments do not reflect real changes in the environment as they ignore interactions between the different variables of the process. Thus, to avoid this problem, the design, optimization and assessment of the process using design of experiment (DOE) seems to be essential^32^. Response surface methodology (RSM) is a mathematical tool to specify the effect of each variable along with their interactions, on the yield of process, and to predict its behavior under a given sets of conditions. The main advantage of the RSM is the reduced number of experiments required to optimize a process. RSM including Central Composite Design (CCD) and Box–Behnken Design (BBD) have been applied to optimize a supercritical fluid extraction (SFE) process. CCD is usually more expensive than BBD due to requires a higher number of runs than BBD^33^.

However, despite the fact that there can be various contaminants that need to be removed to regenerate activated carbons, most of these works investigated polluting substances intentionally added to the activated carbon. To our knowledge this is the first study that propose the use of SC-CO_2_ to regenerate activated carbon used in the gas industry, developing a comprehensive study to optimize and modeling the process. CCD design as a powerful experimental tool was applied to evaluate the influence of operating parameters on the regeneration process. In addition, the process was modeled via mathematical modeling by correlating the experimental data. Moreover, the extract obtained from the SC-CO_2_ method was characterized by GC and the physical properties of activated carbon were studied by SEM and iodine number methods, respectively.

## Material and methods

### Materials

The activated carbon samples used in this study were taken, at summer 2017 during three months, from a gas dehydration unit of the South Zagros Oil and Gas Company (Iran). It should be remarked that, in gas the gas dehydration units activated carbon is used to remove hydrocarbons and aromatic compounds in triethylene glycol. CO_2_ (99.90% purity) was purchased from Aboughadareh Gas Factory.

### Dehydration of natural gas using triethylene glycol

**Figure 1:**
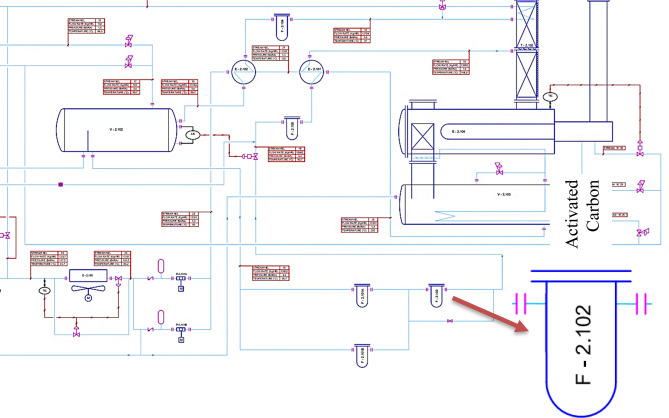
Schematic of unit process.

Raw natural gas is fully saturated with water vapor when produced from an underground reservoir. Because most of the water vapor has to be removed from natural gas before it can be commercially marketed, natural gas is subjected to a dehydration process. One of the most substances used for removing the water from produced gas is glycol. Figure 1 shows a scheme of the typical equipment used for the dehydration process using glycol. While the overall process equipment is similar for all glycol dehydration units, there can be considerable variation among installations. The gas flows through a separator to remove condensed liquids or any solids that might be in the gas. Some absorbers incorporate the separator at its bottom section, in which case the gas then flows upward through a chimney tray into the glycol absorber portion of the vessel. The glycol contactor provides the close contact between the gas and the glycol. The glycol is highly hygroscopic, and most of the water vapor in the gas is absorbed by the glycol. The water-rich glycol is withdrawn from the contactor near the bottom of the vessel above the chimney tray through a liquid level control valve and passes to the regeneration section. The treated gas leaves the contactor at the top through a mist eliminator and usually meets the specified water content. The water-rich glycol can be routed through a heat exchange coil in the top of the reboiler column called the steel column. The heat exchange generates some reflux for the separation of the water from the glycol in the top of the steel and heats the rich glycol somewhat. In some installations, the rich solution passes to a flash tank operating pressure at about 15 to 50 psig, which allows to the absorbed hydrocarbon gas to separate from the glycol. The glycol then flows into the still through an activated carbon filter and a heat exchanger, exchanging heat with the regenerated glycol. Then, it drops through a packed section in the still into the glycol reboiler vessel where it is heated to the necessary high regeneration temperature to near atmospheric pressure. At the high temperature, the glycol loses its ability to hold water; the water is vaporized and leaves through the top of the still. The regenerated glycol flows to the surge tank, from which it is routed through the lean/rich heat exchanger to the glycol pump. The pump boosts the pressure of the lean glycol to the contactor pressure. Prior to entering the contactor, it exchanges heat with the dry gas leaving the contactor or some other heat exchange medium^34^.

### Supercritical fluid extraction method

**Figure 2:**
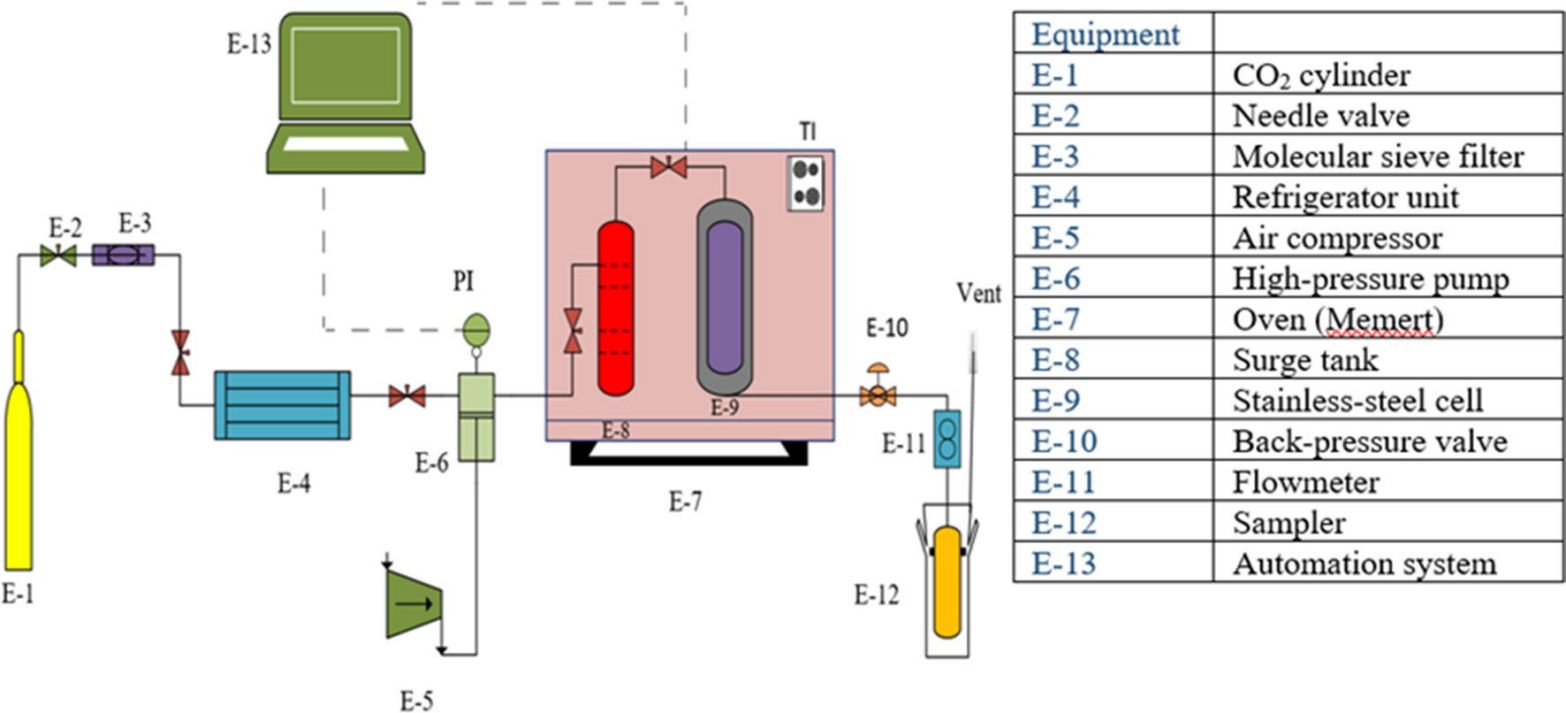
Schematic of Supercritical carbon dioxide extraction.

The main components of the apparatus were a CO_2_ cylinder (E-1), needle valve (E-2), molecular sieve filter (E-3), refrigerator unit (E-4), air compressor (E-5), high-pressure pump (air-driven liquid pump, Haskel, USA) (E-6), oven (Memert) (E-7), Surge tank (E-8), stainless-steel impregnation cell (E-9), back-pressure valve (Xi'an Shelok Instrument Technology Co., Shaanxi, China) (E-10), Flowmeter (E-11), Sampler (E-12), and automation system (E-13). At first, CO_2_ from the reservoir passed through a filter that is rated as a 1µ nominal size to avoid passing any impurity to the process path. Then, CO_2_ was guided towards a refrigerator unit, in which its temperature was drop to -10 °C and 60 Bar. The liquefied CO_2_ was pressurized using a reciprocating pump, and translated towards shell and tube configuration surge Drum. At this drum the fluid temperature was set to proper temperature to reach the supercritical fluid condition. This drum has been designed to reduce the fluctuations and the heat exchange happens by circulating water using 1000 W elements with a temperature accuracy setting of 1 K. A pressure gauge was installed at the output of surge drum to illustrate the outlet fluid pressure. Afterward the fluid was guided towards an extraction cell loaded with 10 g of activated carbon along with glass beads. At the input and output of the extraction cell two filters having Porosity of 1 micron have been installed to prevent the escaping of particles. Existence of glass beads in cell allowed the homogenous distribution of CO_2_ inside the extraction cell. After loading the extraction cell with SC-CO_2_, the cell was set at appropriate temperature and pressure for about 45 min. Following the static stage, the dynamic one begins by opening the sample valve. To prevent the valve from freezing, it was wrapped in a heating element. During the dynamic time, sample collection temperature was kept below 0 °C by using ice booth. Each experiment was done in triplicate^35^. Figure 2 shows the details of supercritical fluid apparatus. The yield of supercritical recovery was calculated through dividing the solute weight per the initial activated carbon. Weight of initial solute in Eq. (1) was determined as follows, first, a known mass of activated carbon was loaded into the filter. Then, after three months, the active carbon is going out from the filter. The difference in the primary and secondary weight of activated carbon indicates the initial value in Eq. (1).

1 $$Yield \,(\% ) = \frac{Weight\,of\,extracted\,solute }{{Weight\,of\, initial\,solute }} \times 100$$

### Design of experiments

Response surface methodology (RSM) was employed to evaluate the effects of pressure (A), temperature (B), CO_2_ flow rate (C) and time (D) on the regeneration yield. The coded and uncoded independent parameters applied in the RSM and their respective levels were tabulated in Table 1. The remaining independent parameters (e.g., particle size and volume of cell) were kept constant during the experimental procedures. The experimental design was based on the central composite design (CCD) using a 30 factorial and star design with six central points as shown in Table 2. Although the cost of this method is more than the Box Behnken method, the authors used this method to evaluate the effect of their work (According to the request of oil industry).

**Table 1:** Un-coded and coded levels of parameters employed in the RSM.

Independent variables	Level of factor				
Coded levels	− 2	− 1	0	1	2
Pressure (A)	100	150	200	250	300
Temperature (B)	313	318	323	328	333
Flow(C)	2	3	4	5	6
Dynamic time (D)	30	60	90	120	150

**Table 2:** Experimental results and predicted yields obtained from RSM.

Run	Pressure (× 1)	Temperature (× 2)	Flow rate (× 3)	Time (× 4)	Yield %	Predicted yield
1.	150	313	3	60	28.73 ± 1.12	28.87
2.	250	313	3	60	48.43 ± 2.18	49.82
3.	150	323	3	60	32.26 ± 2.45	31.56
4.	250	323	3	60	52.88 ± 1.12	51.99
5.	150	313	5	60	45.32 ± 2.16	44.20
6.	250	313	5	60	62.01 ± 1.12	62.18
7.	150	323	5	60	47.49 ± 1.16	46.10
8.	250	323	5	60	63.69 ± 1.29	63.55
9.	150	313	3	120	47.83 ± 1.17	48.65
10.	250	313	3	120	63.26 ± 2.42	64.63
11.	150	323	3	120	52.71 ± 1.62	52.51
12.	250	323	3	120	66.16 ± 2.72	67.96
13.	150	313	5	120	58.88 ± 2.66	59.75
14.	250	313	5	120	71.38 ± 2.45	72.76
15.	150	323	5	120	63.51 ± 1.56	62.81
16.	250	323	5	120	75.45 ± 1.32	75.29
17.	100	318	4	90	35.22 ± 1.23	36.69
18.	300	318	4	90	72.25 ± 2.35	70.12
19.	200	308	4	90	65.62 ± 1.98	63.43
20.	200	328	4	90	67.12 ± 2.39	68.65
21.	200	318	2	90	38.36 ± 1.93	36.83
22.	200	318	6	90	58.62 ± 1.82	59.49
23.	200	318	4	30	35.70 ± 1.18	37.30
24.	200	318	4	150	71.08 ± 1.99	68.82
25.	200	318	4	90	59.54 ± 2.15	59.72
26.	200	318	4	90	57.43 ± 2.17	59.72
27.	200	318	4	90	61.03 ± 2.19	59.72
28.	200	318	4	90	58.54 ± 2.12	59.72
29.	200	318	4	90	61.46 ± 2.22	59.72
30.	200	318	4	90	60.31 ± 2.19	59.72

All experimental runs, which included 8 factorial points, 6 axial points and 5 center points, were based on run order performed. Furthermore, second-order polynomial equation was used to express the yield as a function of the independent parameters.

2 $$\begin{aligned} Y & = \beta_{0} + \beta_{1} A + \beta_{2} B + \beta_{3} C + \beta_{4} D \\ & \quad + \beta_{12} AB + \beta_{13} AC + \beta_{14} AD + \beta_{23} BC + \beta_{24} BD + \beta_{34} CD + \beta_{11} A^{2} + \beta_{22} B^{2} + \beta_{33} C^{2} + \beta_{44} D^{2} \\ \end{aligned}$$

Design expert software (Trial version 7.1) was applied for statistical treatment of the results. Analysis of variance (ANOVA) was employed to determine the statistically significant parameters and interactions using Fisher’s test and its associated probability p(F). The determination coefficients, R_2_, and their adjusted values, R_2,adj_, were used to evaluate the goodness of fit of the regression models.

### Gas chromatography Analysis

A Varian CP-3800 gas chromatograph (Varian instruments) with an FID detector and CP-sil 9 CB capillary column (100 m 0.25 mm, 0.25 μm film thickness) was used to accomplish chemical analysis in this work. The oven temperature was maintained at 35 °C for 7 min before being raised up to 250 °C at 3 °C/min. Injector and detector temperatures were set at 275 °C and 275 °C, respectively. Helium was used as carrier gas at a flow rate of 1.6 ml/min with the samples being injected manually under a split ratio of 1:80. Peaks’ area percentages were used to obtain quantitative data by using of the DHA software.

### Iodine number

The iodine number (IN) was determined according to the ASTM D4607-94 method^36,37^. The iodine number is defined as the milligrams of iodine adsorbed by 1.0 g of carbon when the iodine concentration of the filtrate is 0.02 N (0.02 mol/L). This method is based upon a three-point isotherm. A standard iodine solution is treated with three different weights of activated carbon under specified conditions. The experiment consists of treating the activated carbon sample with 10.0 mL of 5% HCl. This mixture is boiled for 30 s and then cooled. Soon afterwards, 100.0 mL of 0.1 N (0.1 mol L^-1^) iodine solution is added to the mixture and stirred for 30 s. The resulting solution is filtered and 50.0 mL of the filtrate is titrated with 0.1 N (0.1 mol L^-1^) sodium thiosulfate, using starch as indicator. The iodine amount adsorbed per gram of carbon (X/M) is plotted against the iodine concentration in the filtrate (C), using logarithmic axes.

The iodine number is the X/M value when the residual concentration (C) is 0.02 N (0.02 mol.L^-1^). The X/M and C values are calculated by the Eqs. 3 and 4 respectively.

3 $${\text{X}}/{\text{M}} = \left\{ {\left( {{\text{N}}_{1} \times 126.93 \times {\text{V}}_{1} } \right) - \left[ {\left( {{\text{V}}_{1} + {\text{V}}_{{{\text{Hcl}}}} } \right)/{\text{V}}_{{\text{F}}} } \right] \times \left( {{\text{N}}_{{{\text{Na2S2O3}}}} \times 126.93} \right) \times {\text{V}}_{{{\text{Na2S2O3}}}}}\right\}\,/ \,{\text{Mc}}$$

4 $$C = \left( {{\text{N}}_{{{\text{Na2S2O3}}}} \times {\text{V}}_{{{\text{Na2S2O3}}}} } \right)$$

where N_1_ is the iodine solution normality, V_1_ is the added volume of iodine solution, V_HCl_ is the added volume of 5% HCl, VF is the filtrate volume used in titration, N_Na2S2O3_ is the sodium thiosulfate solution normality, V_Na2S2O3_ is the consumed volume of sodium thiosulfate solution and M_C_ is the mass of activated carbon.

### Differential evolution algorithm

Differential evolution algorithm (DEA) is a population-based algorithm like genetic algorithms by similar operators; crossover, mutation, and selection. The main difference in constructing better solutions is that genetic algorithms depend on crossover while DE relies on mutation operation. This main operation is found on the differences of randomly sampled pairs of solutions in the population. The algorithm uses mutation operation as a seek mechanism and selection operation to direct the search toward the probable regions in the search space. The DE algorithm also uses a non-uniform crossover that can take child vector parameters from one parent more often than it does from others. Among the DE’s advantages its simple structure, ease of use, speed, and robustness can be mentioned^38–40^.

## Results and discussion

### Analysis of the response surface design

CCD was used for the design of experiments and process optimization. In this way, four coded operating parameters were defined in five levels, namely − 2, − 1, 0, 1 and 2, to obtain the optimal regeneration yield. Pressure (100–300) bar, temperature (313–333 K), flow rate (2–6 g/min), and dynamic time (30–150 min) were considered as the operating parameters of the process to be studied. Table 2 reports the experiments responses at the different values of operating conditions maintaining constant the particle size at 1 mm. Among models presented in CCD, the quadratic model represents the best model for the prediction and optimization of the regeneration yield. Equation (5) represents the obtained quadratic polynomial model through which the yield was correlated, as a response to the coded independent parameters. Therefore, the RSM predictive model was obtained as follows:

5 $$\begin{aligned} Yield \,(\% ) & = 59.72 + 8.36 A + 1.30 B + 5.67 C + 7.88 D \\ & \quad - 0.13 AB - 0.74 AC - 1.24 AD - 0.20 BC + 0.29 BD - 1.06CD \\ & \quad - 1.58 A^{2} + 1.58 B^{2} - 2.89 C^{2} - 1.66 C^{2} \\ \end{aligned}$$

This model was found to be significant at 95% level of confidence while the corresponding F-value and p-value were calculated to be 92.58 and 0.0001, respectively. Also, accuracy of the model can be tested by investigating the determination coefficient (R_2_). The values of the coefficient of determination (R_2_) and adjusted coefficient of determination (R_2adj_.) were calculated to be 98.86 and 97.79%, respectively. The value of R_2_ indicates a good agreement between the experimental and predicted response values. The value of adjusted R_2_ revealed that only 2.21% of total variations failed to be explained by the model. The lack-of-fit measures the failure of the model to represent data in the experimental domain at points which are not included in the regression. The p-value of the lack-of-fit was higher than 0.05 indicating an excellent fit. Furthermore, Signal-to-noise ratio (SNR) is known as an adequate precision measure. In fact, SNR compares the range of predicted values at design points to the average prediction error. As a SNR greater than 4 represents desirable results, the achieved ratio (35.30) served as an adequate signal as it was much smaller than the actual effect size. Low C.V. (3.32%) indicates the reliability of the carried-out experiments. The ANOVA of quadratic model was carried out using the data tabulated in Table 3 with the purpose of examining significance of the variables as linear, quadratic and interaction coefficients of the RSM. Those variables and their interactions with higher regression coefficient and smaller p-value (p < 0.05) have a significant influence on the regeneration yield^41,42^. Analysis results reported in Table 3, as well as, the parameters which statistically indicated highly significant impact on the yield were linear term of pressure, CO_2_ flow rate and time (p < 0.0001), followed using the quadratic term of flow rate(p < 0.0001). The quadratic term of time (p = 0.0003), the linear term of temperature (p = 0.0037) as well as the quadratic term of pressure and temperature (p = 0.0005), represent significant influence on the yield.

**Table 3:** ANOVA of RSM model.

Source	Sum of squares	df	Mean-square	F-value	*p* value	Significance
Model	5657.71	14	404.12	92.58	< 0.0001	Significant
X_1_	2116.69	1	2116.69	484.93	< 0.0001	Significant
X_2_	51.6	1	51.60	11.82	0.0037	Significant
X_3_	972.44	1	972.44	222.78	< 0.0001	Significant
X_4_	1881.69	1	1881.69	431.09	< 0.0001	Significant
X_1_* X_2_	0.35	1	0.35	0.080	0.7806	Insignificant
X_1_* X_3_	11.17	1	11.17	2.56	0.1305	Insignificant
X_1_* X_4_	31.22	1	31.22	7.15	0.0173	Significant
X_2_* X_3_	0.81	1	0.81	0.19	0.6719	Insignificant
X_2_* X_4_	1.70	1	1.70	0.39	0.5424	Insignificant
X_3_* X_4_	22.63	1	22.63	5.19	0.0379	Significant
X1* X1	86.35	1	86.35	19.78	0.0005	Significant
X2* X2	86.49	1	86.49	19.81	0.0005	Significant
X3* X3	289.36	1	289.36	66.29	< 0.0001	Significant
X4* X4	95.97	1	95.97	21.99	0.0003	Significant
Residual	65.47	15	4.36			
Lack of fit	50.65	10	5.06	1.71	0.2884	Insignificant

### Effect of process parameters on regeneration yield

In this section the effect of the different operational parameters on the supercritical regeneration of activated carbon were examined with three dimensional graphs, while the other variables were maintained at its respective fixed middle level(Pressure, 200 bar, Temperature, 318 K, flow rate,4 g/min and time 90 min), corresponding to zero code.

**Figure 3:**
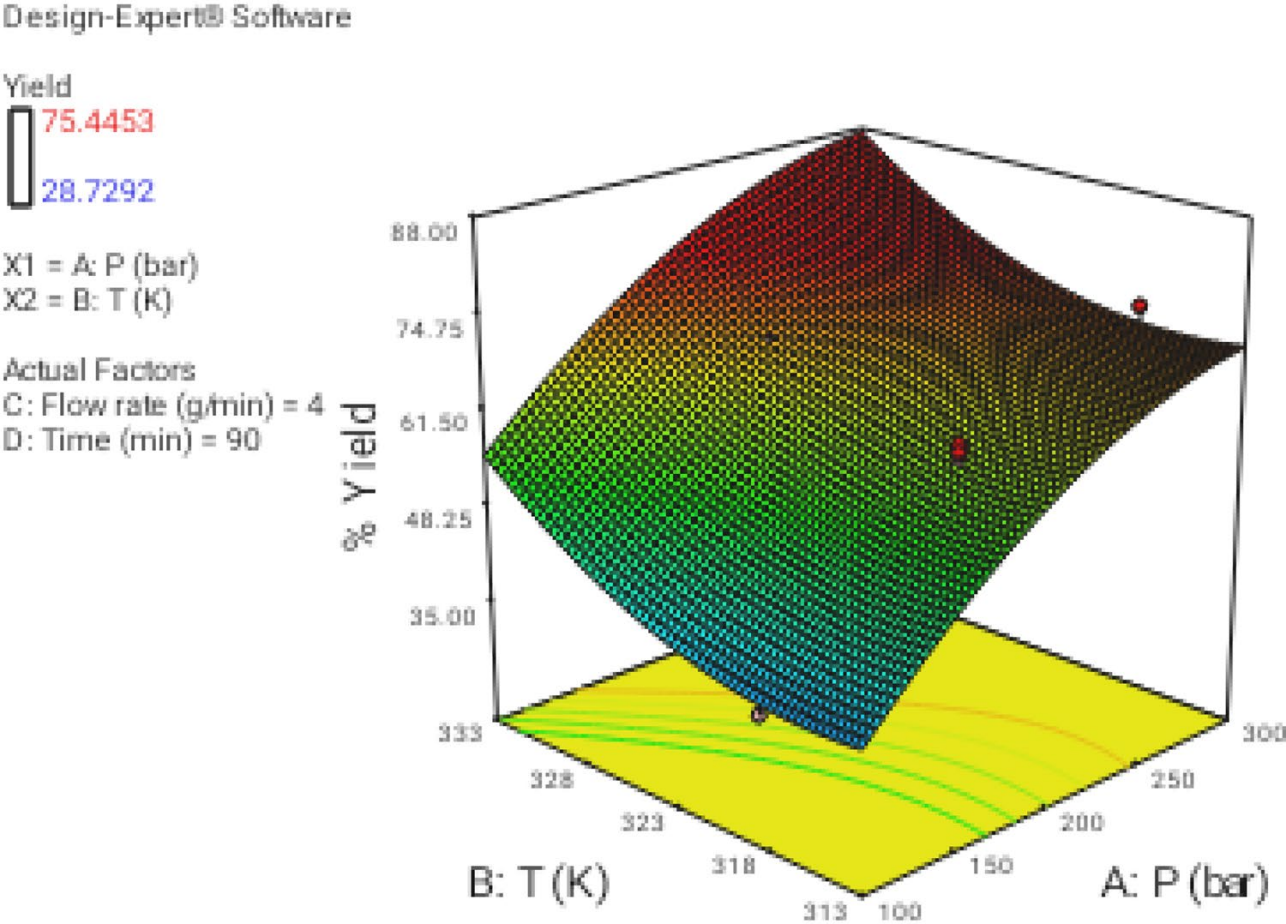
3D graphic surface for the effects of temperature and pressure on the yield.

**Figure 4:**
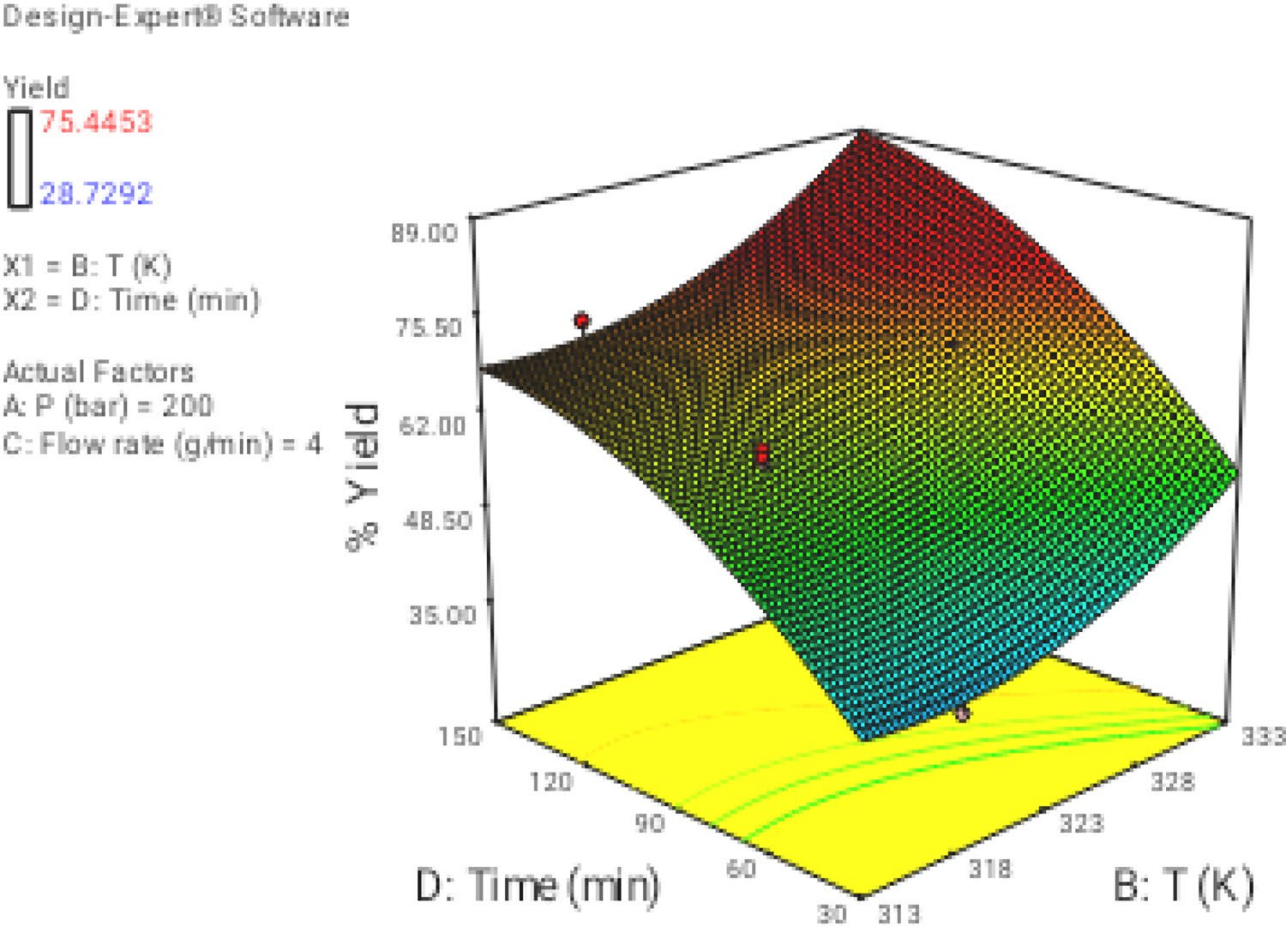
3D graphic surface for the effects of temperature and time on the yield.

**Figure 5:**
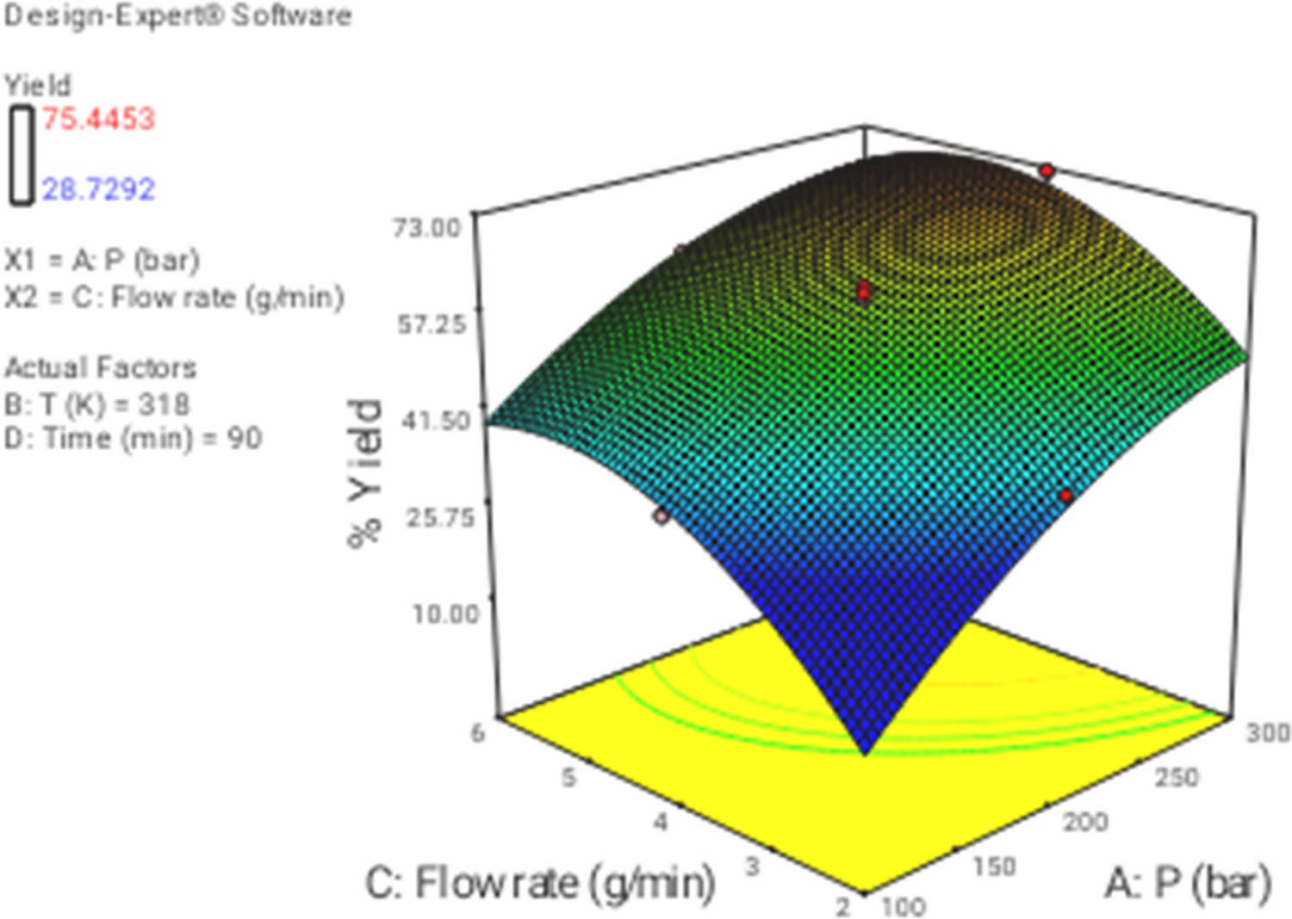
3D graphic surface for the effects of flow and pressure on the yield.

**Figure 6:**
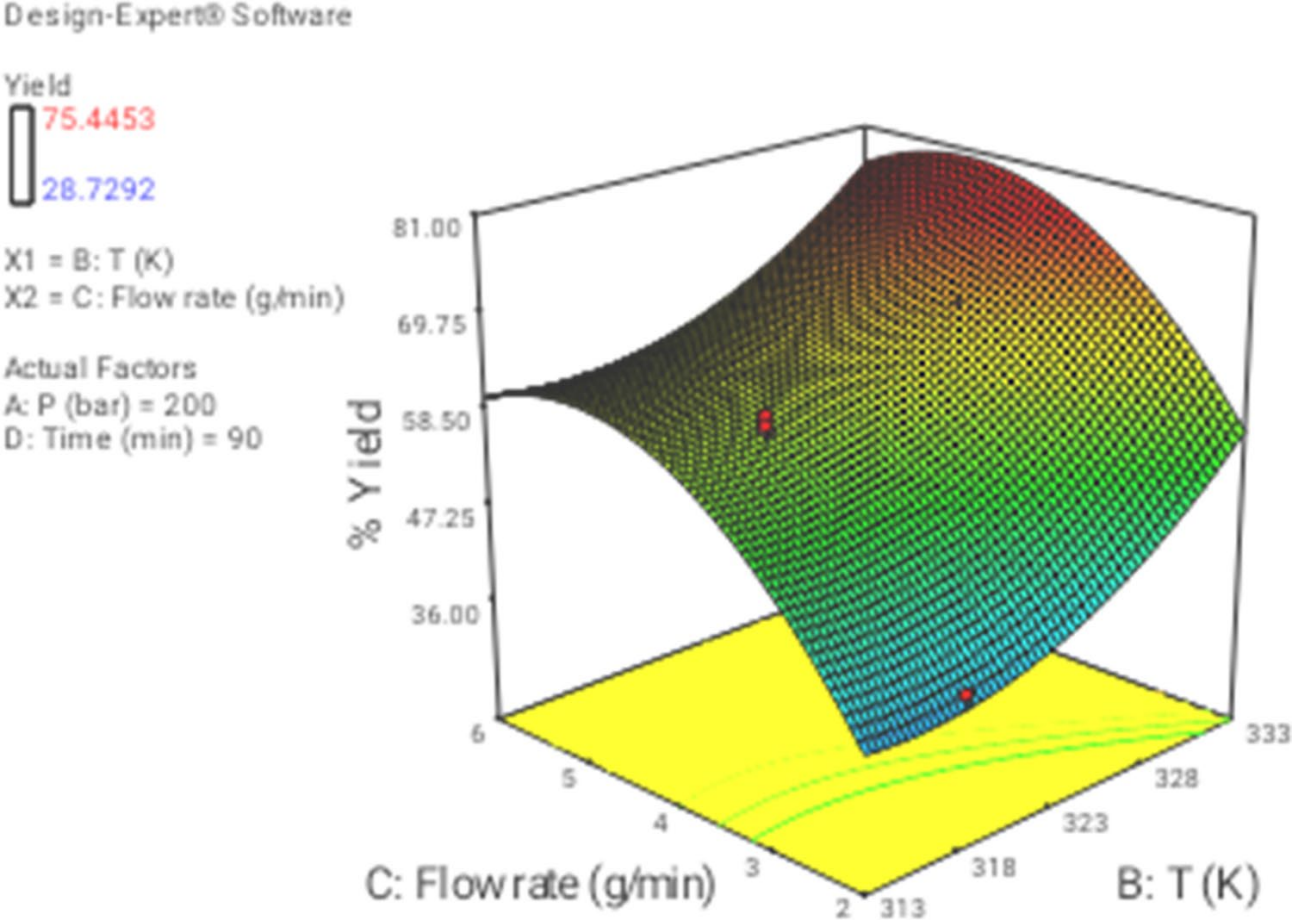
3D graphic surface for the effects of temperature and flow on the yield.

Figure 3 shows the 3D plot for the influence of pressure and temperature. It can be observed that the regeneration yield increased with increasing pressure and temperature from 100 to 300 bar and from 313 to 333 K, respectively. Based on CCD, pressure had the most important influence on the regeneration yield. Particularly, the increase of pressure leads to increase solvent power of CO_2_ and therefore solubility of the solutes increased as well^9,43^. On another hand, the increase in temperature had a slight positive effect on the regeneration yield (Fig. 4). At a pressure of 100 to 300 bar, with the increase in temperature, the solubility of solute increases, and it is due to the increment of the solute vapor pressure effect. therefore, the solubility of solute increases with the increase in temperature. On the other hand, the extract vapor pressure is raised with increasing temperature, which leads to an enhancement in the supercritical fluid diffusivity. Figure 5 denotes the 3D plot of the yield as a function of pressure and flow rate at 323 K and 90 min. Increasing the CO_2_ flow rate from 1 (code =  − 2) to 4 g/min (code = 1) reduces the film thickness around the particles leading to lower resistance for mass transfer around the particles and accordingly enhancing the regeneration of the activated carbon. Whereas at more than 4 g/min with reduction of residence time (SC-CO_2_- particles contact time) a negative effect of flow rate was observed on the regeneration yield. This contrast effect was also observed in Figs. 6, 8 and 9.

**Figure 7:**
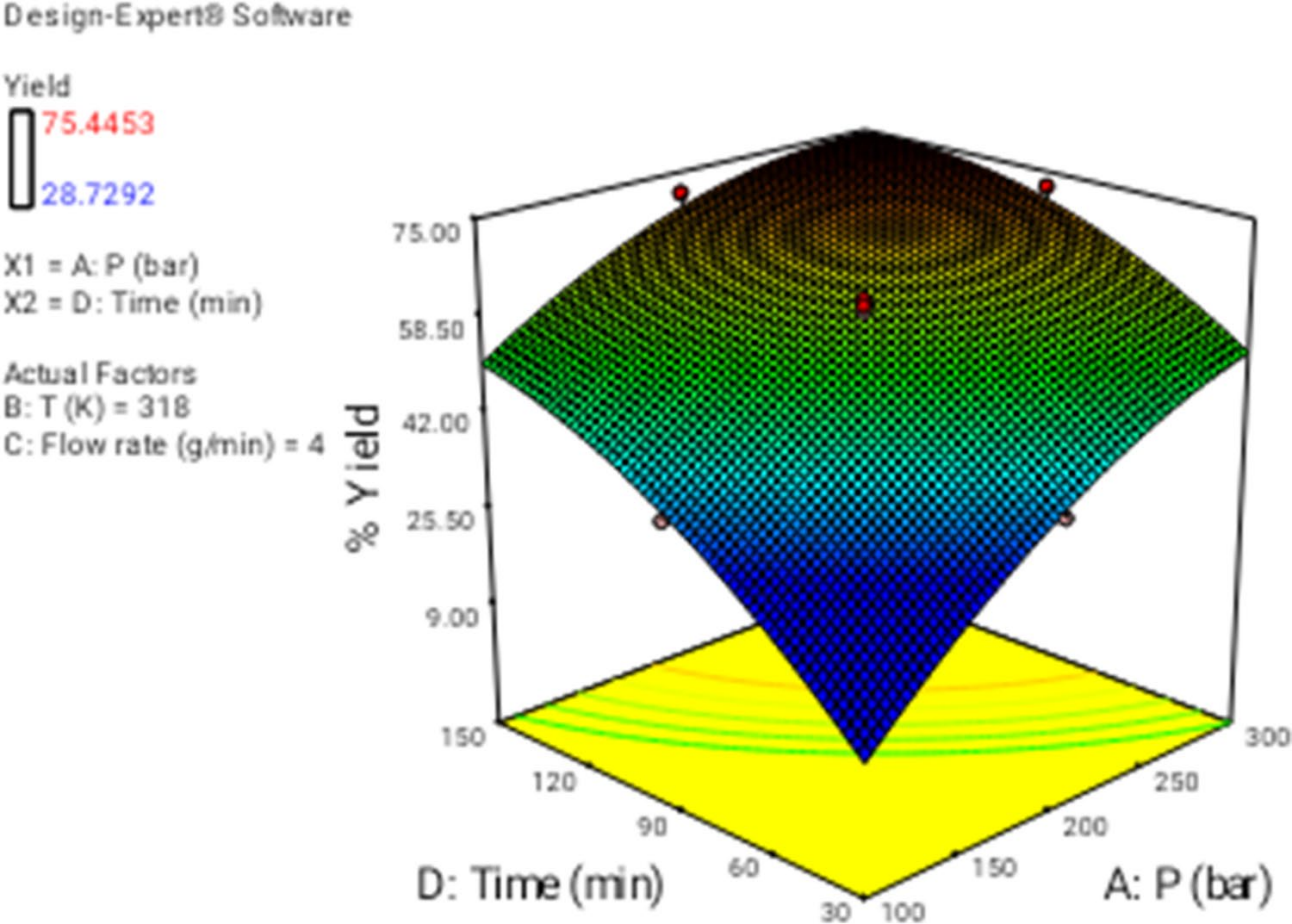
3D graphic surface for the effects of pressure and time on the yield.

**Figure 8:**
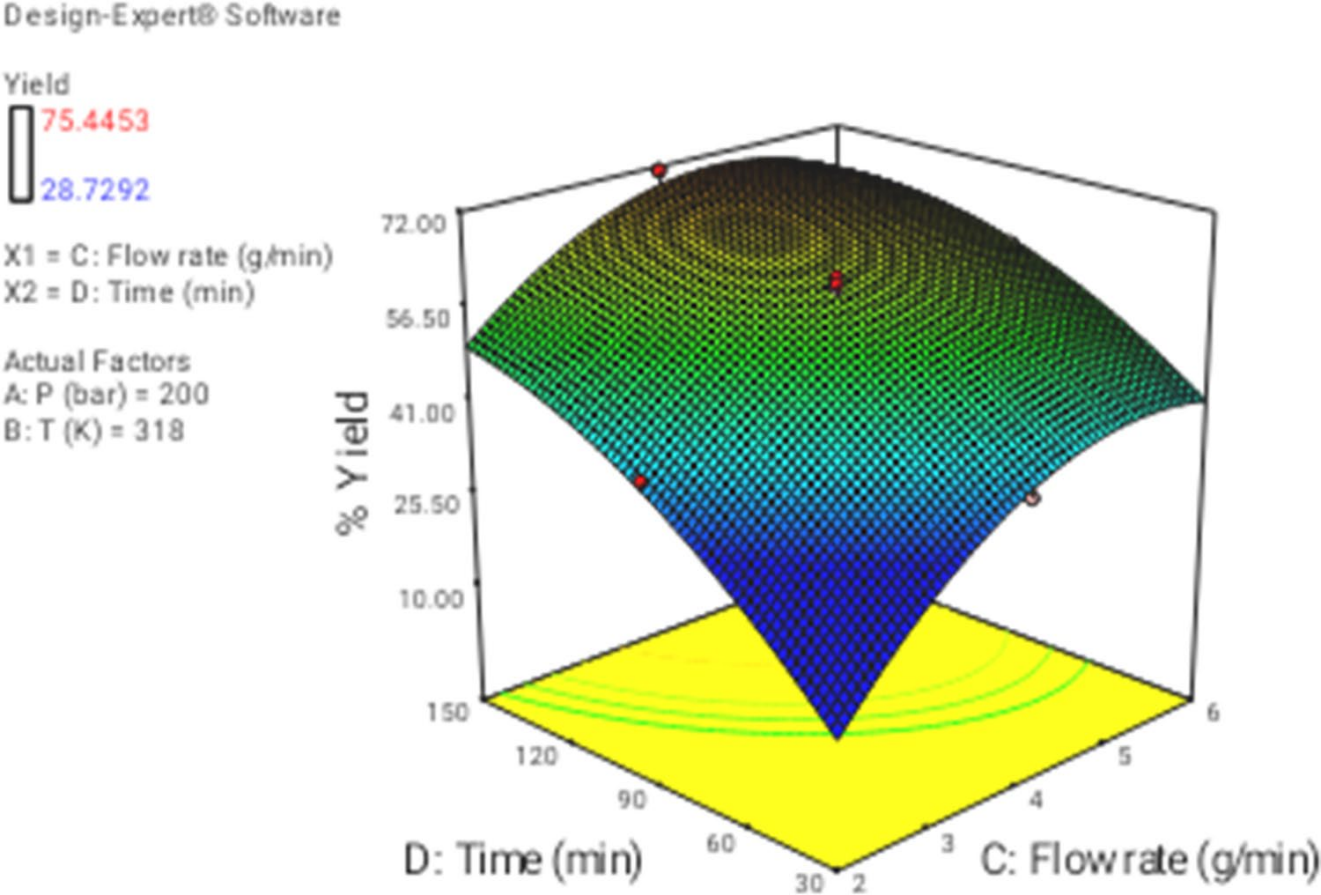
3D graphic surface for the effects of flow and time on the yield.

**Figure 9:**
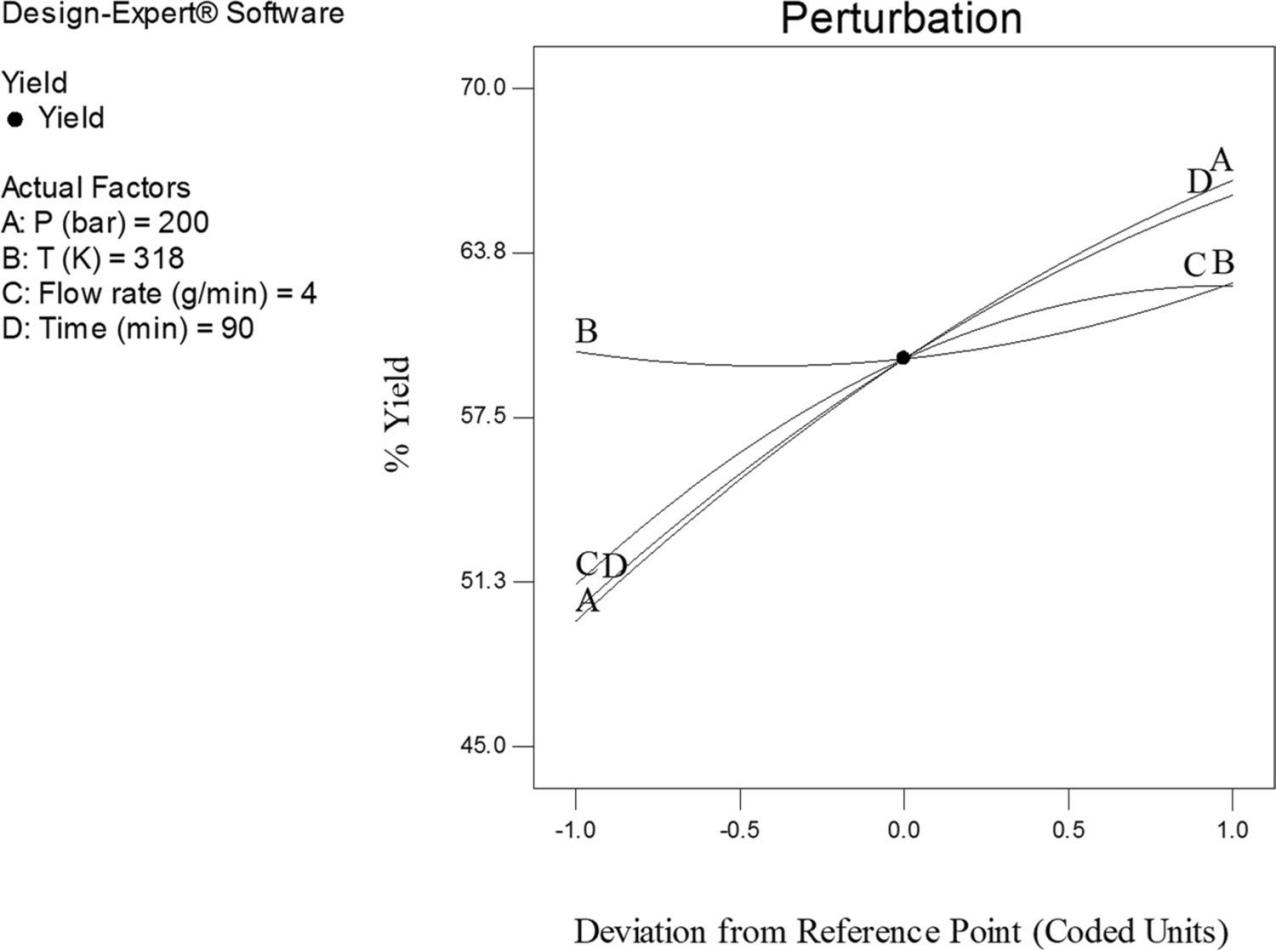
Perturbation plot of operational parameters obtained through CCD on the yield.

Figures 4, 7, and 8 illustrate the effect of dynamic time and flow rate on the regeneration yield. As can be seen in Figs. 4, 7, and 8 the regeneration yields gradually increase with increasing the dynamic time, achieving its highest value about 150 min. This behavior has been reported by other researchers and explained in terms of the increase of the ratio between SC-CO_2_ and the solute as the dynamic time increase^35^. On another hand, the residence time decrease as flow rate increase, while the external mass transfer coefficient increased, so these opposite phenomena canceled their effects out leading to the almost constant yield. Moreover, the increase of the flow rate could reduce the contact time between the solvent and the activated carbon. This phenomenon could be related to the channeling effect, where SC-CO_2_ at high flow rates would just flow around the samples with no ability to diffuse through the pores within the samples. Furthermore, the increased flow rate could cause the sample compaction limiting the amount of CO_2_ able to being in direct contact with the sample matrix. Similar results were reported by some researchers^33,44–48^. In addition, the perturbation plot (Fig. 9) revealed the significant effect of all process variables on the extraction. A perturbation plot does not show the effect of interactions and it is like one factor-at-a-time experimentation. The perturbation plot helps to compare the effect of all independent variables at a particular point in the design space. The response is plotted by changing only one factor over its range while holding the other factors constant.

The optimal conditions to obtain the highest yield of regeneration from activated carbon were determined at 285 bar, 333 K, 4 g/min, and 147 min and the predicted yield was 93.75%. Average actual yield (94.25%) was in good agreement with the estimated value, revealing the ability of the developed model in terms of extraction process prediction and optimization.

### Gas chromatography analysis

The solute composition determined for the extract, at the optimum condition, by gaseous chromatography (GC) were shown in Table 4. Thirty-two components were identified in the extract obtained using SC-CO_2_ which comprised 95.2% of the extract. The main components extracted by SC-CO_2_ were N-C6 (4.90), N-C7 (9.45%), N-C8 (9.04%), N-C9 (11.90%), N-C10 (12.40%) and N-C11 (4.73%).

**Table 4:** Results of gas chromatography.

Number	Compound	Retention time	Area %
1.	C3	5.971	2.3
2.	IC4	6.337	2.6
3.	NC4	6.654	4.2
4.	IC5	7.810	3.3
5.	NC5	8.437	4.4
6.	2,2-Di methyl butane	9.62	0.5
7.	2,3-Di methyl butane	10.875	0.3
8.	2-methyl pentane	11.15	0.5
9.	3-methyl pentane	11.96	0.7
10.	N-C6	13.094	4.9
11.	2,2-Di methyl pentane	15.22	2.1
12.	2,4-Di methyl pentane	15.57	0.15
13.	benzene	17.48	0.14
14.	2-methyl-hexane	19.58	0.36
15.	2,3-Di methyl pentane	19.74	0.36
16.	3-methyl hexane	21.36	0.46
17.	TAME	21.71	0.23
18.	2,2-dimethyl hexane	22.06	0.36
19.	N-C7	23.89	9.45
20.	methyl cyclo hexane	26.56	8.2
21.	unknown	31.91	2.1
22.	toluene	34.31	3.3
23.	N-C8	40.29	9.93
24.	ethyl benzene	44.23	1.2
25.	p-xylene	45.30	0.45
26.	m-xylene	45.83	0.4
27.	o-xylene	46.30	0.38
28.	N-C9	55.47	11.9
29.	unknown	59.86	1.3
30.	N-C10	67.25	12.4
31.	1,2,3-tri methyl benzene	77.14	2.1
32.	N-C11	77.93	4.73
33.	Total		95.2

### Iodine results

Iodine number is a measure of iodine molecules adsorbed in the pores of a particle, which indicates the pore volume capacity and extent of micro pore distribution in the activated carbon. Iodine number can be correlated with the adsorbent ability to adsorb low molecular weight substances. Iodine adsorbed results in extracted and non-extracted activated carbon samples were shown in Table 5. Results of physical and adsorptive characteristics denote that SC-CO_2_ appears to be a suitable way for the regeneration of activated carbon.

**Table 5:** Results of iodine number test.

	Sample		
	Original	Un-process	Process
Iodine number	930 mg/g	230 mg/g	910 mg/g

### Evaluating the structure of activated carbon

**Figure 10:**
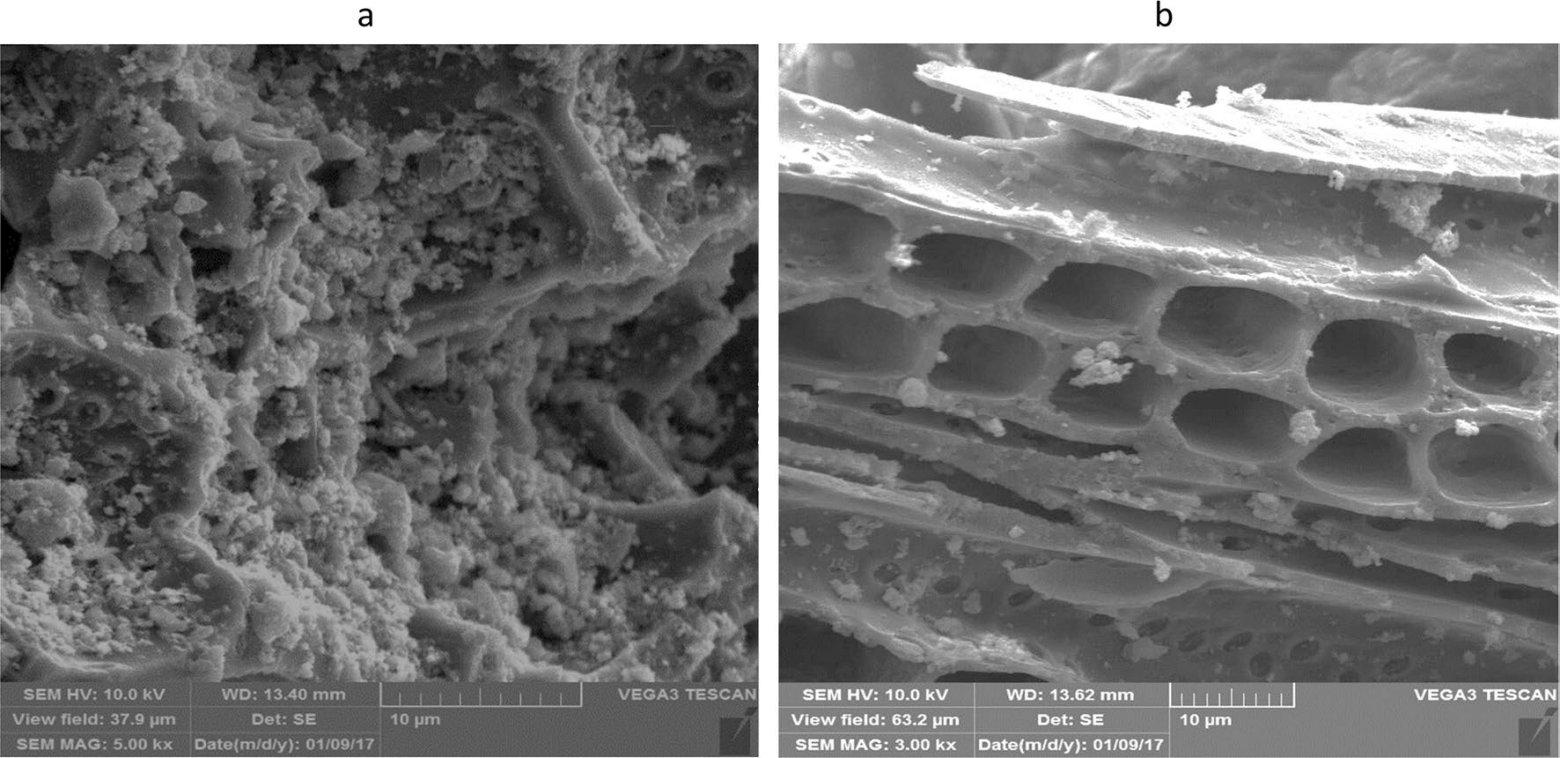
(**a**,**b**) SEM image of (**a**) un-process, (**b**) process of activated carbon.

As was previously mentioned the structure and surface morphology of non-extracted and extracted activated carbon samples was evaluated by scanning electron microscopy (SEM). Figure 10a,b presents two micrographs of activated carbon before (a) and after supercritical regeneration (b). The micrograph of the unprocessed sample shows an uneven and rough surface covered uniformly with a layer of solute. The structure of the treated sample is more porous and the surface is clearly deflated and depleted of solute due to the high recovery efficiency obtained by the extraction with supercritical carbon dioxide.

### Mathematical modeling

The experimental desorption was correlated with a kinetic model proposed by Tan and Liou^17,49^. This model assumes linear desorption kinetics in the adsorbed phase, and has a high applicability for correlating desorption behavior using SC-CO_2_ with only one fitting parameter. The following assumptions were considered in the formulation of the model:

1. The system was isothermal and isobaric.
2. The physical properties of the SC-CO_2_ were constant during the extraction process.
3. The extraction model was expressed as an irreversible desorption process.
4. All particles were considered to be spherical and the solutes were uniformly distributed in their structures.
5. The volume of the solid matrix (particles) was not changed during the extraction process.
6. The solvent flow rate was constant along the bed and uniformly distributed without radial dispersion.
7. Axial dispersion was neglected

**Figure 11:**
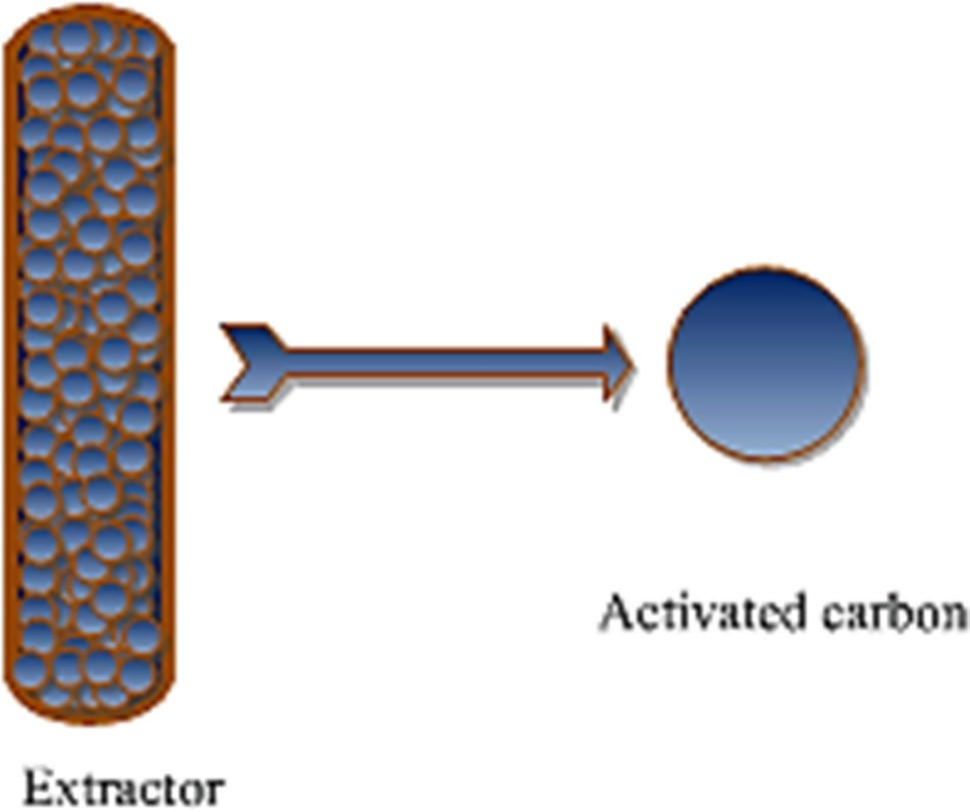
A schematic diagram of the particles and bed.

A schematic diagram of the particles and bed was presented in Fig. 11. According to the above assumptions, the material balances for the solute in the solid and bulk phases are as follows:

Bulk phase:

6 $$\varepsilon \frac{\partial C}{{\partial t}} + u\frac{\partial C}{{\partial z}} = - \left( {1 - \varepsilon } \right)\frac{{\partial C_{P} }}{\partial t}$$

7 $$C = 0 \to \left( {t = 0, z} \right)$$

8 $$C = 0 \to \left( { z = 0, t } \right)$$

Solid phase:

9 $$\frac{{\partial C_{P} }}{\partial t} = - KC_{P}$$

10 $$C_{P} = C_{P0} \to \left( {t = 0} \right)$$

The concentration at the exit of the packed bed can then be obtained by:

11 $$C_{e} = C_{P0} .\frac{1 - \varepsilon }{\varepsilon }\left\{ {exp\left[ { - k\left( {t - \frac{\varepsilon L}{u}} \right)} \right] - exp\left( { - kt} \right)} \right\}$$

K is the adjustable parameter of the kinetic model.

The adjustable parameter was computed by minimizing the errors between experimental and calculated yield values. The average absolute relative deviation (AARD), described using the following equation was applied to evaluate the adjustable parameter:

12 $$AARD\% = \frac{1}{N}\mathop \sum \limits_{i = 1}^{N} \left( {\left| {\frac{{y_{i,cal} - y_{i,exp} }}{{y_{i,exp} }}} \right|} \right) \times 100$$

**Figure 12:**
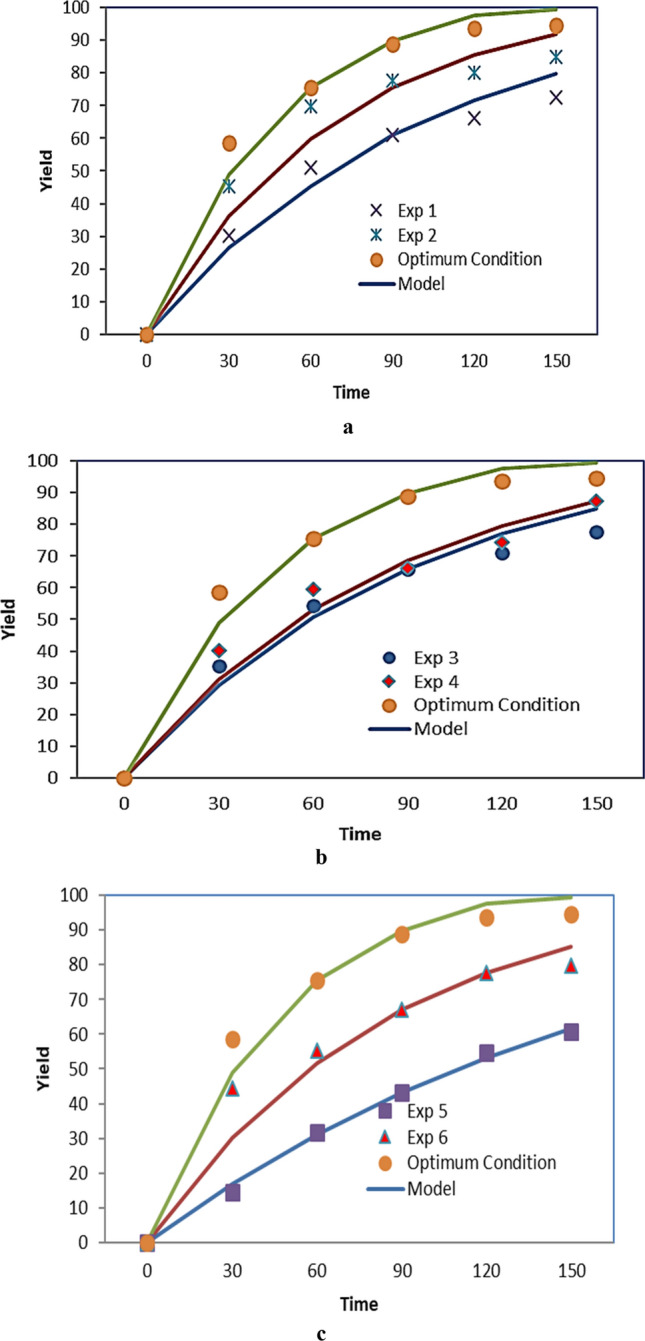
Effect of process parameters on the yield of extraction versus time.

The mathematical modeling was applied to study the impact of pressure, temperature and flow rate as on the regeneration process. Figure 12 and Table 6 show the modeling results by using the kinetics model. Fig 12a shows a positive effect of the pressure on the regeneration process at fixed temperature, flow rate, particle size and dynamic time. This effect was related to the increase of the SC-CO_2_ density as pressure increase. As was expected, the increase of density leads to an increase in the solubility of the solutes in SC-CO_2_. On the other side, increasing pressure decreased the diffusion coefficient of CO_2_. The decreasing effect of mass transfer coefficient may lead decreasing the yield. Nevertheless, the effect of increasing density and solubility overcomes the decreasing effect of diffusivity on the final yield of extraction^22,50^. The adjustable parameter of the model (K) and AARD values for the different extraction runs were reported in Table 6. The effect of temperature on the regeneration yield of activated carbon was shown in Fig. 12b. The yield increased with increasing the temperature at fixed pressure and flow rate. As previously mentioned, this may be due to the increase of the vapor pressure of the solute. In the present section, the effect of temperature on the regeneration yield at points of 318, 328 K and optimum temperature were modeled. The flow rate of carbon dioxide was another parameter that was investigated. The experimental data and results of model in three flow rates including 1, 2 and 4 (optimum) were shown in Fig. 12. Based on the results indicated in Fig. 12c, the yield was higher at optimal points. As can be seen in Fig. 12 the experimental data were well described with the kinetics model. Values of average absolute relative deviation are in the range 5.91-9.04%.

**Table 6:** Results of model.

Exp	P (bar)	T (K)	Q (g/min)	Time (min)	K	% AARD
1.	150	318	5	150	0.0100	7.90
2.	250	318	5	150	0.0142	5.80
3.	250	318	3	150	0.0108	8.23
4.	250	328	3	150	0.0116	8.73
5.	200	323	2	150	0.0058	4.59
6.	200	323	4	150	0.0115	9.04
7.	285	333	4	147	0.0205	5.91

### A single‑sphere model (SSM)

Single sphere model (SSM) proposed by Crank^51^ with an assumption that particle size is one of the main factors on the diffusivity step in the extraction process. In addition, diffusion is assumed to occur in the sphere surface area of a particle solute. Dissemination of SC-CO2 in sphere equations is conducted to determine the diffusiveness of solvent to dissolve in matrices. Other assumptions made for an SSM model are: (1) The resistance of mass transfer is zero, (2) all of the particle sizes are homogenous, (3) the main factor in the extraction process is intraparticle of mass transfer, (4) the solute is in the inert porous sphere, and lastly, (5) all of the solutes in the bed will be extracted, and the extracted component will be dissolved in the particles by a process similar to diffusion. Equation 13 exhibits the diffusion equation for a constant coefficient.

13 $$Y = \frac{{M_{t} }}{{M_{\infty } }} = 1 - \frac{6}{{\pi^{2} }} \mathop \sum \limits_{n - 1}^{\infty } \frac{1}{{n^{2} }}exp\frac{{D_{eff } t \pi^{2} n^{2} }}{{R^{2} }}$$

where Mt is the total amount of the diffusing substances entered on the sheet at a specific time, M∞ is the particular quantity after countless time, *D*_*eff*_ is the diffusivity, R is the radius of particle solute, and t is the time.

The single sphere model is usually applied to determine the diffusivity coefficient and mass transfer between solvent and solute. Compared to another kinetic model, the single sphere model is easily applied, particularly the shrinking core model^52^ and the broken intact cell^53^ due to one adjustable parameter. This is because one adjustable parameter is fit enough to determine the mass transfer process of experimental data. Furthermore, a single-sphere model can assess the effect of the parameter on the diffusivity process between solvent and solute^54^.

**Figure 13:**
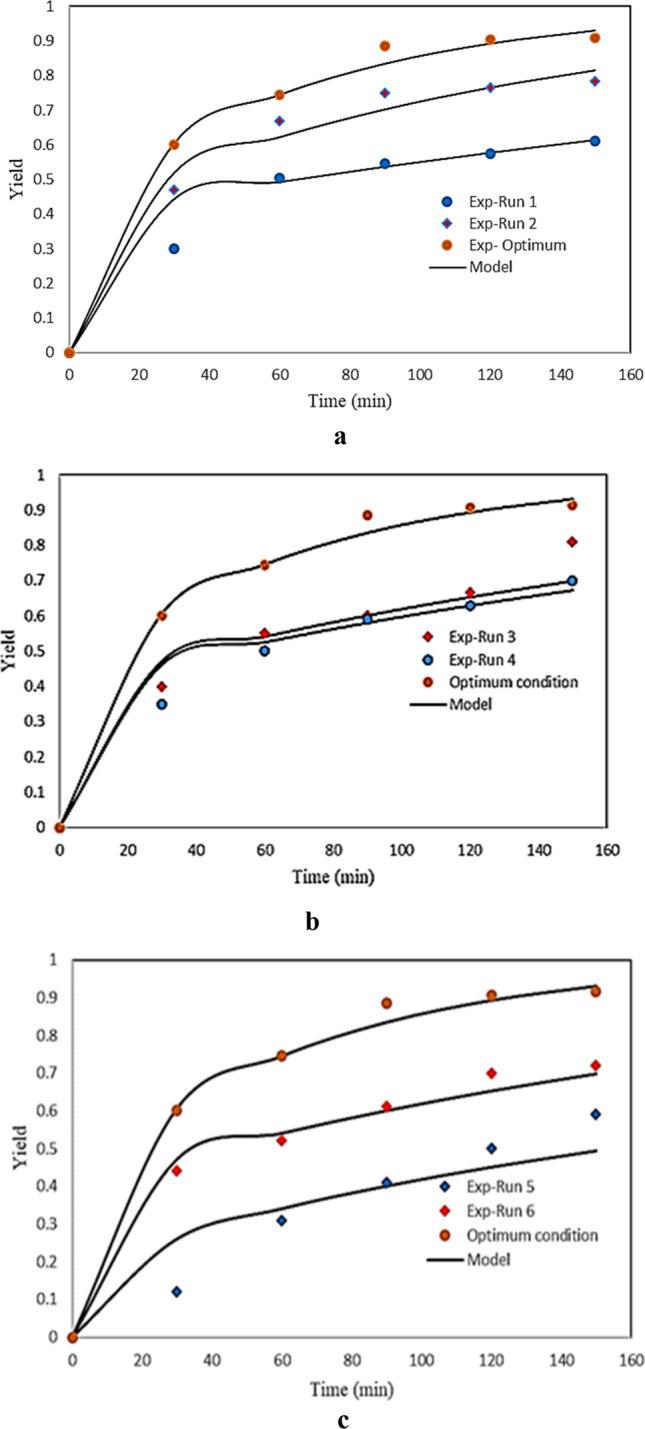
Effect of process parameters on the yield of extraction versus time.

The single sphere model was fitted to the experimental data with effective diffusivity as a fitting parameter, using average absolute deviation (AARD). Table 7 shows the AARD and effective diffusivity. The minimum %AARD was 2.08% at 285 bar and 333 K, while the maximum %AARD was 19.04% at 200 bar and 323 K. As shown in Fig. 13 the model successfully fitted the exponential trends of the extraction process. Putra et al.^1^ applied single sphere model for fitting the experimental data of modified supercritical carbon dioxide with error below 5% with the highest diffusivity coefficient was 6.794 × 10^‐12^ m^2^/s at operating condition was 10 MPa, 40 °C and particle size 425 µm. Moreover, Aris et al.^2^ determined Momordica charantia extract yield with different mean particle size as well as diffusion coefficient, De, in the extraction process with and without co-extractant. Based on the results, mean particle size of 0.3 mm gave the highest extract yield, 3.32% and 1.34% with and without co-extractant respectively. Whereas, the value of De at 0.3 mm mean particle size, with and without co-extractant are 8.820 × 10^−12^ and 7.920 × 10^−12^ m^2^/s respectively.

**Table 7:** Results of single sphere model.

Exp	P (bar)	T (K)	Q (g/min)	Time (min)	${D}_{eff}$×10^–12^	% AARD
1.	150	318	5	150	2.88	10.60
2.	250	318	5	150	7.53	5.80
3.	250	318	3	150	4.42	7.83
4.	250	328	3	150	5.93	8.73
5.	200	323	2	150	2.91	10.39
6.	200	323	4	150	2.87	19.04
7.	285	333	4	147	13.81	2.81

## Conclusion

Response surface methodology (RSM) was used in order to find out the effect of pressure, temperature, flow rate and dynamic time on the regeneration yield of activated carbon using SC-CO_2_. The optimal conditions for regeneration were found at 285 bar, 333 K, 5 g/min and 147 min which resulted in a regeneration yield equal to 93.71%. The regeneration effectiveness was revealed by the iodine number and visualized by SEM micrographs. The kinetic models proposed by Crank and Tan et al. successfully described the regeneration process using SC-CO_2_ and the estimated regeneration yields using the model adequately agreed with the experimental data for all studied conditions. The model parameters (K) and effective diffusivity was obtained by best fitting procedure applying differential evolution (DE) algorithm. Gas chromatography analysis was performed to identify the composition of solutes at the optimal extraction condition.

## Data Availability

All data are available within the published paper.

## List of symbols

Cal: Refers to calculated
C: Concentration of the solute in Co_2_ (mol/m^3^)
$Ce$: Exit concentration of solute (mol/m^3^)
*D*_*eff*_: Effective diffusivity of solute (m^2^/s)
dp: Particle size (mm)
exp: Refers to experimental
K: Adjustable parameter
$L$: Length of extractor (m)
N: Number of data
P: Pressure (bar)
$Q_{{{\text{co}}2}}$: Mass flow rate of CO_2_ (kg/s)
R: Radius of particle
$$t$$: Time (s)
T: Temperature (K)
$$u$$: Solvent velocity (m/s)
Y: Yield
z: Axial position in column

## Greek letters

$$\rho$$: Density (kg/m^3^)
$$\varepsilon$$: Bed porosity
