# Publisher Correction: Separation of hydrocarbons from activated carbon as a porous substance in a glycol regeneration process using supercritical carbon dioxide

**DOI:** 10.1038/s41598-022-26230-x

**Published:** 2022-12-14

**Authors:** Mohammad Najafi, Zahra Arab Abousadi, Bizhan Honarvar, Seyed Ali Sajadian

**Affiliations:** 1 Department of Chemical Engineering, Islamic Azad University of Marvdasht, Marvdasht, Iran; 2 South Zagros Oil and Gas Production, National Iranian Oil Company, Shiraz, 7135717991 Iran; 3 Department of Chemical Engineering, Faculty of Engineering, University of Kashan, Kashan, 87317‑53153 Iran

Correction to: *Scientific Reports* https://doi.org/10.1038/s41598-022-23722-8, published online 19 November 2022

The original version of this Article contained an error in Reference 22, which was incorrectly given as:

“Sodeifian, G. & Sajadian, S. A. CO2 utilization as gas antisolvent for the pharmaceutical micro and nanoparticle production: A review. *Arab. J. Chem.*, 104164 (2016).”

The correct Reference is listed below.

“Esfandiari, N. & Sajadian, S. A. CO_2_ utilization as gas antisolvent for the pharmaceutical micro and nanoparticle production: A review. *Arab. J. Chem.*, 104164 (2022).”

The original Article has been corrected.

